# South-to-south mentoring as a vehicle for implementing sustainable health security in Africa

**DOI:** 10.1186/s42522-021-00050-x

**Published:** 2021-10-06

**Authors:** Stephanie Marie Norlock, Patrick W. Okanya, Anastasia Trataris, Michael E. Hildebrand, Jean de Dieu Baziki, Imane Belkourati, Maureen Ellis

**Affiliations:** 1 International Federation of Biosafety Associations (IFBA), Ottawa, Canada; 2 Carleton University, Ottawa, Canada; 3 Technical University of Kenya, Nairobi, Kenya; 4 National Institute for Communicable Diseases (NICD), Johannesburg, South Africa; 5 African Union - Pan African Veterinary Vaccine Centre (AU-PANVAC), Debre Zeit, Ethiopia; 6 MCI Santé Animale, Mohammedia, Morocco

**Keywords:** Global mentorship, South-to-south, OneHealth, Biosafety, Biosecurity, Global health security, Frontline workers

## Abstract

**Background:**

While sustainability has become a universal precept in the development of global health security systems, supporting policies often lack mechanisms to drive policies into regular practice. ‘On-paper’ norms and regulations are to a great extent upheld by frontline workers who often lack the opportunity to communicate their first-hand experiences to decisionmakers; their role is an often overlooked, yet crucial, aspect of a sustainable global health security landscape. Initiatives and programs developing transdisciplinary professional skills support the increased bidirectional dialogue between these frontline workers and key policy- and decisionmakers which may sustainably narrow the gap between global health security policy design and implementation.

**Methods:**

The International Federation of Biosafety Associations’ (IFBA) Global Mentorship Program recruits biosafety and biosecurity champions across Africa to provide local peer mentorship to developing professionals in their geographic region. Mentors and mentees complete structured one year program cycles, where they are provided with written overviews of monthly discussion topics, and attend optional virtual interactive activities. Feedback from African participants of the 2019–2020 program cycle was collected using a virtual Exit Survey, where aspects of program impact and structure were assessed.

**Results:**

Following its initial call for applications, the IFBA Global Mentorship Program received considerable interest from professionals across the African continent, particularly in East and North Africa. The pilot program cycle matched a total of 62 individuals from an array of professional disciplines across several regions, 40 of which were located on the African continent. The resulting mentorship pairs shared knowledge, skills, and experiences towards translating policy objectives to action on the front lines. Mentorship pairs embraced multidisciplinary approaches to harmonize health security strategies across the human and animal health sectors. South-to-South mentorship therefore provided mentees with locally relevant support critical to translation of best technical practices to local capacity and work.

**Conclusion:**

The IFBA’s South-to-South Global Mentorship Program has demonstrated its ability to form crucial links between frontline biosafety professionals, laboratory workers, and policy- and decision-makers across several implicated sectors. By supporting regionally relevant peer mentorship programs, the gap between health security policy development and implementation may be narrowed.

**Supplementary Information:**

The online version contains supplementary material available at 10.1186/s42522-021-00050-x.

## Background

### The Global Health security implementation gap

While there has been significant progress in the development of biosafety and biosecurity guidelines, policy frameworks, and corresponding legislation on national and global scales, their implementation remains a challenge. The prevention and mitigation of biological risk, ranging from unintentional disease outbreak to the deliberate proliferation of biological weaponry, is a dynamic and diverse set of systems and strategies that, while often rigorously designed, falls short when it fails to translate from paper to regular practice over the long term. Although the sometimes short half-lives of certain policies and programming may be considered an issue of sustainability, it is proposed that this issue of longevity is a symptom of a greater systemic implementation gap in global health security. This implementation gap between policy design and execution results in a unidirectional flow of information from policy- and decisionmakers to the front-line workers who regularly handle biological materials and are responsible for the promotion and maintenance of the organizational cultures necessary for sustainable biosecurity systems [1]. This prescriptive method aiming to ‘trickle down’ best practices to those at the laboratory bench has resulted in workers at the front line often being ill-equipped to flexibly incorporate policies that they are tasked to implement, and without adequate agency to provide feedback when applying these standards in their regular work. Conversely, many policy- and decisionmakers at global and even national levels remain inadequately informed of the local and human contexts of the work and environments that they regulate. As multilateral and multisectoral dialogues surrounding global health security continue to acknowledge and promote the value of a combined top-down and bottom-up approach to policy design [2–4], policy implementation must effectively follow suit through collaborative methods encouraging bidirectional communication between frontline laboratory workers and policy- and decisionmakers. Open, inclusive dialogue across all implicated sectors and levels of governance may optimize and align the divergent and duplicated approaches to common issues in global health security produced by professional siloes. In this way, evolving best practices in global health security may not only include all implicated professional fields in their design, but they may also encourage the common acceptance of responsibility and accountability for maintaining health security standards [5].

### Transdisciplinary skills within the health security workforce

A necessary first step in the implementation of a collaborative, multidisciplinary health security workforce is refining the collective, transdisciplinary set of soft skills, often referred to as human skills, across relevant professional fields [6]. While specific terminology surrounding the concept of human skills in this regard may sometimes include technical training and refer to overall competency of facility staff, it often also includes factors such as attitude towards and broader general experience with consideration and implementation of biosafety and biosecurity standards [7]. Serving as a crucial counterpart to the technical understanding of biological materials and related emerging technologies, the acknowledgement and fostering of these relevant human skills such as risk communication and critical thinking enables the collaborative interdisciplinary work required to begin narrowing, and eventually bridging, the implementation gap. This transnational adoption of new norms across entire health security workforces could appear unrealistic if not for the considerable discussions already occurring on the topic. The ubiquitous OneHealth approach is a primary example of how interdisciplinary work may be conducted to address multifaceted issues in the life sciences and related professional fields. OneHealth acknowledges the interconnectedness of human health, animal health, and the environment as an integrative approach as opposed to distinct, specific fields of study [8]. Since its inception in 2007 as an interfacing tool across animal and public health, the OneHealth approach includes the use of human skills which transcend discipline-specific technical knowledge [6]. Overarching themes of these competencies include leadership and management skills, comprehension and application of values and ethics, as well as systems thinking as it pertains to the implication of all collaborating professional disciplines on a given issue. This approach was further embraced by international organizations such as the World Health Organization (WHO), the Food and Agriculture Organization of the United Nations (FAO), the World Organization for Animal Health (OIE), and others to develop a Laboratory Leadership Competency Framework for the sustainable development of national health laboratory systems [9], which specifically includes one overarching competency dedicated to biosafety and biosecurity. The robust framework defines mastery of competencies in degrees of ability to apply technical knowledge with the use of human skills, such as progression from ability to describe a standard to the ability to implement or even evaluate it in different contexts. As such, this framework may provide indicators for the professional development of laboratory leaders developing competency through anticipated complementary implementation initiatives. Both OneHealth and Laboratory Leadership Competency Frameworks illustrate the quality of human skills required for an effective multidisciplinary workforce in the life sciences and beyond in their designs, and point towards opportunity for specific application to biosafety and biosecurity personnel.

When specifically addressing the global biosecurity landscape, there has been broad, intensive discussion regarding the cultivation of global biosafety and biosecurity culture and code of conduct by platforms such as the United Nations Biological Weapons Convention Meeting of Experts, the Global Health Security Agenda, and the G7 Global Partnership Against the Spread of Weapons and Materials of Mass Destruction [10, 11]. As this code of conduct and an integrative definition of biosafety and biosecurity culture are still emerging [11], there is considerable need and opportunity for continued and further contributions from frontline biosafety and biosecurity workers in both writing these standards as well as assessing their application to regular work.

### Mentorship as an integrative health security implementation tool

Mentorship is a practice of professional development which is consistently recognized as valuable by academic and other professional sectors in its contribution to intergenerational knowledge and career development in youth [12]. Despite this, it remains underprioritized by leadership across the life and health sciences [13–15], and is consequently underutilized across relevant sectors. Mentorship combines development of knowledge and understanding of a discipline with personal, future-oriented support, usually for young or otherwise developing professionals. With much emphasis being placed on the demonstration of technical competency, biosafety and biosecurity professional development has largely consisted of technical training, limited postsecondary education programs, and certification initiatives [11, 16, 17]. As historically conducted training and certification programs do not address the human element of biosafety and biosecurity on a personal level, there has been little opportunity to develop and exercise the aforementioned human skills critical for the development of the next generation of biosafety and biosecurity professionals. It is proposed that mentorship in biosafety and biosecurity disciplines is an effective mechanism to develop these skills in tandem with training and certification methods. Following the discussed OneHealth and Laboratory Leadership frameworks, mentorship may serve as a subsequent implementor to develop and normalize outlined competencies in the workplace. The effective implementation of biosafety and biosecurity professional development which addresses both technical and human elements may contribute to an integrative normalization of best practices in preventing and mitigating biological risk at and beyond the laboratory bench.

Where technical biosafety and biosecurity professional development is at times geographically dependent due to national and regional guidelines and legislation, mentorship needs and style similarly differ depending on sociocultural and professional factors [12]. Local biosafety and biosecurity mentorship, where mentees have access to mentors within their geographic region, may achieve a higher degree of understanding through shared personal and professional experiences. Understanding is also shared across factors such as gender, socioeconomic background, and age. Local mentorship may not only effectively address global policies and practices, but regional and national ones as well. This is particularly important when addressing low- and middle-income countries, where biosafety and biosecurity capacity can be quite limited, and there is considerable need for continued local leadership in these disciplines. The African continent is one example, where Joint External Evaluations (and lack thereof), demonstrate extremely limited health security capacity across several regions [18]. Although mentorship appears to be the most cost-effective and accessible professional development method when compared to technical training and certification, mainstream understanding of its best practices is often biased to high-income country settings [12]. Institutionally, it is reported that emerging life and health science students and young professionals in low- and middle-income countries struggle to find formalized or even available mentorship despite the demand for it [12, 14, 19, 20]. Although this has resulted in well executed North-South twinning and general mentorship programming in biosafety and biosecurity disciplines [21], this method does not ultimately achieve the development and maintenance of local professional networks over the long term. As local South-to-South mentorship contributes to local competency, professional networks, and future policy implementation, it may be argued that it more sustainably contributes to the capacity of resource-limited settings in comparison to North-South twinning and mentorship.

Local mentorship in biosafety and biosecurity provides mentees with culturally and regionally relevant professional development, where technical concepts are discussed within the context of national and regional systems and legislation. Local mentorship can similarly connect mentees to local policy- and decision-makers through emphasis on national and regional organizations and professional aggregates such as professional biosafety associations. Further, the program is equipped to directly include policy- and decision-makers as mentors or mentees in the program, resulting in direct matches with frontline professionals. As such, this approach complements traditional technical training and certification programs while providing frontline workers with valuable context pertaining to and linkages with governance and influential professional aggregates in their country or region, including local policy implementation gaps. In this way, local mentorship in biosafety and biosecurity aims to address the needs for further dialogue between frontline workers and policy- and decision-making members of local biosafety and biosecurity landscapes. It additionally sustains these professional populations and their respective contributions over the long term in accordance with an increasingly diverse professional arena in the regional and global health security fields. Otherwise stated, local mentorship in biosafety and biosecurity is proposed to narrow the implementation gap in global health security as an accessible professional development method that incorporates the often overlooked, yet critical, human elements of mitigating biological risk necessary for the dynamic process of delivering policy into consistent practice.

The International Federation of Biosafety Associations’ Global Mentorship Program (IGMP) is a unique global South-to-South biosafety and biosecurity mentorship program to be offered to the global professional community. Developed in 2019, its pilot program cycle (2019–2020) primarily consisted of, and was greatly influenced by biosafety and biosecurity professionals from across the African continent. Following the successful completion of this pilot program cycle in early 2020, IGMP presents as a novel ongoing vehicle for health security policy implementation. IGMP serves as a key policy implementor through the empowerment of local biosafety and biosecurity professionals, facilitation and contribution to local professional networks, and direct engagement with national, regional, and global leaders in health security.

## Methods

### Participant recruitment

The International Federation of Biosafety Associations (IFBA) is a not-for-profit, non-governmental organization composed of a federation of professional associations with a mandate to manage biological risks. This federation’s professional network includes biosafety officers, laboratory technicians, research scientists, academics, policymakers, and other professionals that contribute to the organization’s mission of, ‘safe, secure, and responsible work with biological materials’. The IFBA is composed of 47 national and regional Member Biosafety Associations, as well as like-minded Observer Organizations, across Asia, Africa, Europe, the Americas, and Oceania, resulting in a truly global community of biosafety and biosecurity professionals. IFBA Member Associations include national and regional biosafety associations, often composed of biosafety and biosecurity professionals from multiple institutions across human, animal, and environmental sectors. IFBA Observer Associations are stakeholder organizations which include international organizations, non-governmental organizations, government agencies, and other relevant multisectoral professional associations. The professional and overall diversity of this global community has provided opportunity for the development of truly representative global programming,

IGMP was initially advertised to the IFBA’s global network of Certified Professionals, other past participants of the IFBA Professional Certification Program [22, 23], as well as to the members and affiliates of IFBA Member and Observer Associations. Global Mentors were defined as experienced biosafety and biosecurity professionals with at least 3–5 years of professional experience, and at least two IFBA Professional Certifications. IFBA Global Mentees were defined as emerging professionals in biosafety and biosecurity, without any specific professional prerequisites, but who had interest in eventually pursuing an IFBA Professional Certification. Particular emphasis was placed on the accessibility of the program, where all program content was available virtually, and with no associated cost to participants. Open-ended application questions for mentors and mentees focused on topics such as prior experience with professional mentorship, communication and leadership skills, as well as future goals related to biosafety and biosecurity professions. Applicants were not specifically assessed for technical knowledge in biosafety and biosecurity, given the certification requirement for mentors. Successful IFBA Global Mentor and IFBA Global Mentee applications were selected based upon quality of written responses with regards to understanding the role of mentorship as opposed to technical training programming, detailed descriptions of the applicant’s professional goals and background relevant to regional and global biosafety and biosecurity, as well as descriptions of specific interests within these disciplines (e.g. general biorisk management, biowaste management, biosecurity). Program cycle cohorts were developed to be as geographically balanced as possible to encourage interregional dialogue across mentorship pairs during interactive activities, as well as encourage global exposure of IGMP as a developing program across implicated stakeholder groups within IFBA’s federation and beyond.

IFBA Global Mentors and IFBA Global Mentees were matched based upon shared geographic region, gender, and professional discipline when possible. In addition to local matching, there was particular focus on gender matching women mentees with experienced women mentors within their region. Matching within institutions was discouraged to maximize the expansion of mentees’ professional networks in biosafety and biosecurity. Once matched, mentors and mentees communicated on an informal basis as was convenient for them, but were encouraged to match the pace of monthly program material distribution. Although the program was delivered virtually, local matching did provide the opportunity for mentors and mentees to meet in person if they resided in the same city, or to jointly attend national or regional biosafety and biosecurity events as members of their national or regional biosafety association. The pilot 2019–2020 program cycle was conducted over email correspondence, and has since evolved to be hosted on an interactive online platform during the 2020–2021 program cycle.

### Program delivery

IGMP’s 2019–2020 program cycle was delivered with the intention of being open-ended and flexible to suit the needs of its program participants. Given that this program cycle was a pilot phase, the primary focus during program delivery was to determine the amount of support required for program participants, including facilitation of mentee-mentor matching and provision of relevant technical resources. Technical discussion point newsletters were distributed each month to program participants, and included topics and resources relating to foundational and trending topics in biosafety and biosecurity. These topics specifically included those relevant to global health security, including overviews of foundational biosecurity terms (e.g. insider threats, defining biosecurity risks as opposed to biosafety risks), health security decision-making mechanisms (e.g. the Global Health Security Agenda, the Biological Weapons Convention), as well as discussions surrounding relevant soft skills development (e.g. risk communication, professional networking, and the development of local biosafety culture). With the onset of the global COVID-19 pandemic in early 2020, there was an increased promotion of local risk assessment approaches, as well as shared national, regional, and global guidelines with regards to safe handling and work with the SARS-CoV-2 virus and related pathogenic materials.

The main indicator of progress in the program was the timely submission of Progress Reports, where mentor and mentee pairs were asked to complete a brief, survey-style report form using an online survey platform. These reports aim to summarize the discussions and activities that mentorship pairs took part in throughout the program cycle, and were an opportunity to share local challenges and experiences that contextualize national and regional biosafety and biosecurity systems. Mentee and mentor pairs were expected to complete a minimum of four brief Progress Reports per program cycle, resulting in 1 report every three months, however, pairs were free to complete as many Progress Reports as they wished. At the end of the program cycle, participants were invited to complete an Exit Survey, the results of which have helped steer the subsequent cycle of IGMP. Questions regarding program impact included the following, where participants rated their agreement with each statement on a seven point Likert scale (1 being strong disagreement with a statement, and 7 being strong agreement with a statement):

*PI1. The information I learned has strengthened my ability to implement sustainable risk-based biosafety and biosecurity measures.*

*PI2. The IFBA Global Mentorship Program helped me connect, interact, and share best practices with other biosafety/biosecurity professionals in my region.*

*PI3. Participating in the IFBA Global Mentorship Program has provided me with a better understanding of global health security priorities and initiatives.*

*PI4. I am interested in further engaging with my regional/national biosafety association.*

*PI5. IGMP has helped me develop leadership and/or networking skills to encourage others and grow the biosafety/biosecurity profession in my region.*

*PI6. I feel I am in a better position to interact with decisionmakers and contribute to my national biosafety and biosecurity policies and/or strategies.*

Additionally, the Exit Survey included self-reported attendance of optional activities delivered virtually during the 2019–2020 program cycle through multiple choice style questions. The survey also collected qualitative data through an open-ended comment section for general feedback, as well as experience with their mentor or mentee.

During the 2019–2020 program cycle, two optional activities were hosted online via teleconferencing. The first activity was a round-table discussion regarding open-access biosafety and biosecurity resources, which ultimately constructed the outline of an online open-access biosafety and biosecurity e-library. Participants discussed resource accessibility, and existing resource repositories which either worked for them, or had noticeable gaps which could be filled using a new, harmonized tool. The second optional activity was a webinar presentation featuring the IFBA Executive Director with regards to narrowing the implementation gap in global health security.

Following the completion of the 2019–2020 program cycle, IGMP and lessons learned from its pilot phase were presented at the 6th World One Health Congress in October 2020 by members of the IFBA Secretariat and ongoing IGMP participants at a session entitled *Empowering Global Health Security and Policy in Africa* [24]. This focus on the large proportion of African participants and corresponding perspectives of the 2019–2020 IGMP program cycle inspired further investigation of program outcomes specific to implicated African regions.

## Results

### 2019–2020 program cycle participant recruitment

The pilot program cycle application period launched in February 2019, and attracted over 130 mentee applications, as well as over 40 mentor applications, demonstrating a high degree of interest from the global IFBA Certified Professional community for a comprehensive, structured mentorship program. For mentee applications, regions with the highest level of engagement included East Africa, West Africa, Middle East and North Africa (MENA), and South Asia (specifically, Pakistan). Unfortunately, due to the lack of IFBA Certified Professionals with at least two certifications in the West African region at that time, there were limited opportunities for within-region matching for the 2019–2020 program cycle. The 2019–2020 program cycle of IGMP achieved an approximately 60% gender match between mentors and mentees. When gender matches were available within a mentee’s region, they were pursued for the particular purpose of providing women mentees in biosafety and biosecurity with the option to experience mentorship with an experienced, local woman biosafety or biosecurity professional.

**Fig. 1:**
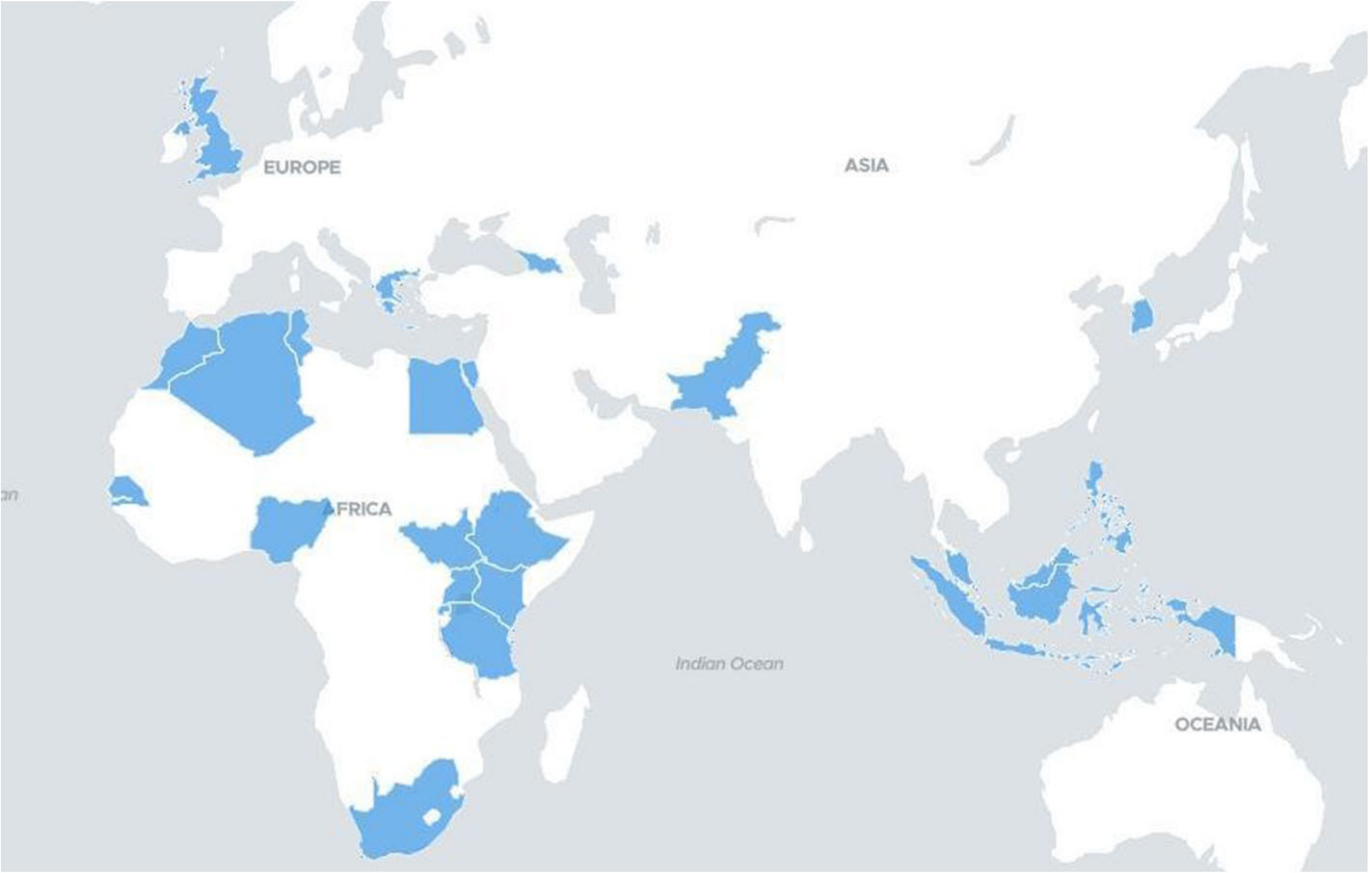
IFBA Global Mentorship Program 2019–2020 program participants geographic distribution (total n = 62). Participants reported residence across 22 countries (Algeria = 8; Egypt = 4; Ethiopia = 1; Georgia = 1; Greece = 1; Indonesia = 1; Kenya = 10; Malaysia = 3; Morocco = 5; Nigeria = 4; Pakistan = 10; Philippines = 1; Rwanda = 1; Senegal = 1; Singapore = 1; South Africa = 1; South Korea = 1; South Sudan = 1; Tanzania = 1; Tunisia = 2; Uganda = 3; United Kingdom = 1)

Sixty-two participants from 22 different countries were officially recruited for the 2019–2020 IGMP program cycle (Fig. 1). Incongruences between regional volumes of mentor and mentee applications were mitigated through out of region matches; the 2019–2020 program cycle achieved an 85% within-region rate overall, where all within-region mentorship pairs were engaged in South-to-South local mentorship. Of the 62 recruited participants, 40 participants (18 mentors, 22 mentees) from 13 countries were located on the African continent.

### 2019–2020 program cycle Progress report and exit survey results

Of the 40 African participants who participated in the 2019–2020 program cycle, 26 completed the program cycle exit survey, resulting in a 65% response rate for this group. Program participation, measured through Progress Report submission and Optional Activity attendance, as well as program impact measured through Exit Survey results were analyzed across these 26 participants. The demographics of survey respondents were relatively similar to the demographic breakdown of the 2019–2020 cohort across gender, mentorship role, and geographic region (Table 1). Of these 26 participants who completed the Exit Survey, the mean number of Progress Report submissions within their respective mentorship pairs was observed to be approximately 4.82, which is almost one additional report per program cycle over the minimum submission rate.

**Table 1:** IGMP 2019–2020 exit survey respondent characteristics across the African continent (n = 26)

Variable	n(%)
**Gender**	
**Man**	18 (69.2%)
**Woman**	8 (30.8%)
**Role**	
**Mentor**	11 (42.3%)
**Mentee**	15 (57.7%)
**Region**	
**North Africa**	11 (42.3%)
**East Africa**	12 (46.2%)
**West/Southern Africa**	3 (11.5%)
**Gender-Matched Pairs**	
**Yes**	16 (61.5%)
**No**	10 (38.5%)
**Region-Matched Pairs**	
**Yes**	22 (84.6%)
**No**	4 (15.4%)

Survey respondents provided feedback on IGMP’s pilot program cycle through closed multiple choice and Liker t-scale style questions, as well as open short answer questions to gather qualitative information regarding program experience. When African respondents were grouped regionally, the majority were either located in North Africa (Algeria, Morocco, Tunisia, Egypt) or East Africa (Kenya, Tanzania, Ethiopia, Rwanda, South Sudan), with only one mentee from South Africa and two mentees from West Africa (Nigeria, Senegal) being grouped together in a combined category. These mentees, as well as one outlying East African mentee, compose the minority of respondents who were not matched with a mentor within their geographic region. The overall feedback from African survey respondents was positive regarding program impact, where overall average Likert-scale responses indicate agreement to strong agreement with impact statements (Overall Likert = 6.58 +/− 0.07).

**Fig. 2:**
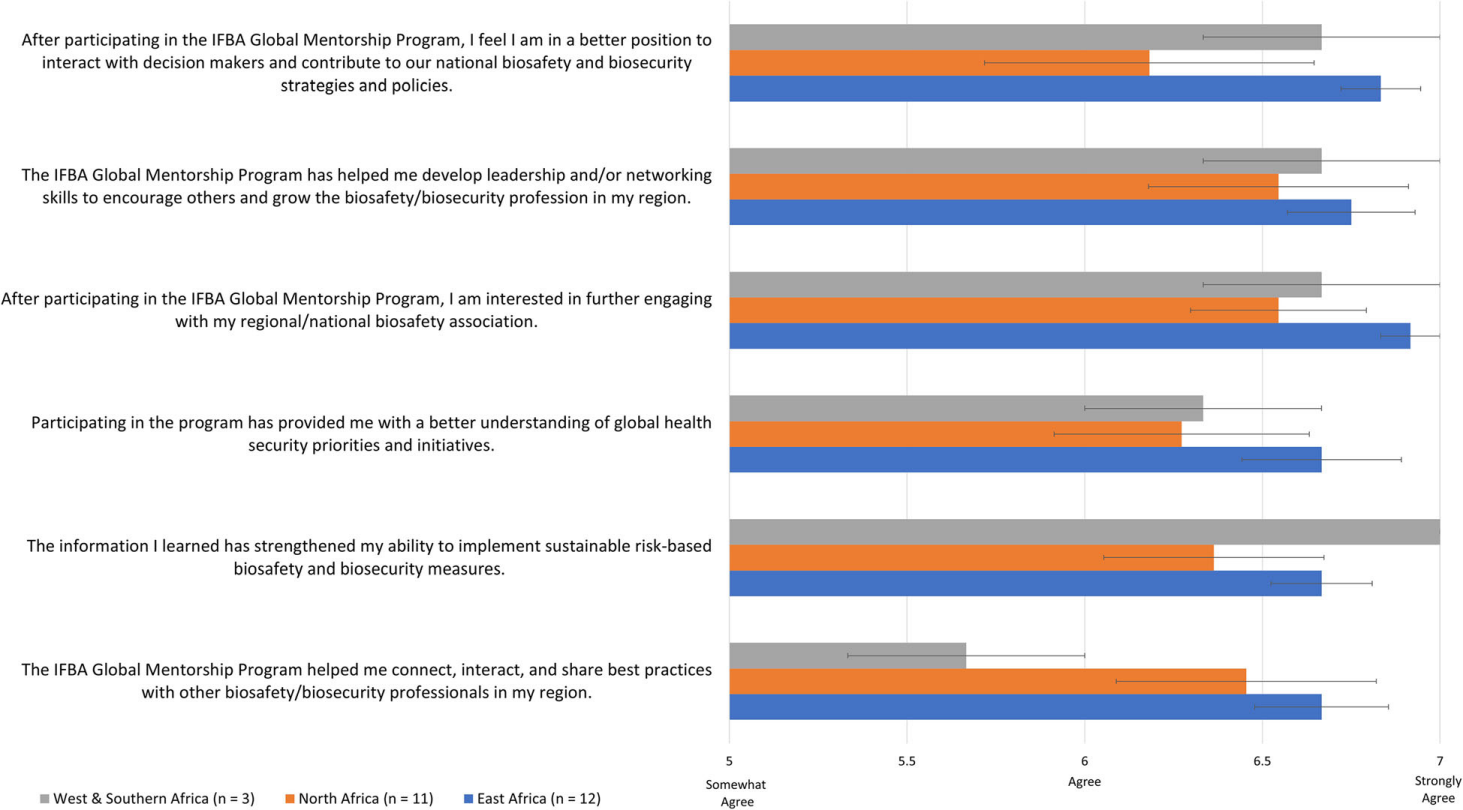
Average exit survey program impact response scores across regions, with standard error. Response scored based upon a 7-point Likert scale, with 1 being strong disagreement with a statement, and 7 being strong agreement

Across regions, program participants indicated that IGMP has contributed to their knowledge and understanding of best technical practices, including their application to broader global contexts relevant to biosafety, biosecurity, and overarching health security (Fig. 2). Despite receiving mentorship outside of their region, all mentees in the small West and Southern African group (Fig. 2, grey bars) strongly agreed that information provided by IGMP strengthened their ability to implement sustainable risk-based biosafety and biosecurity measures (7.00 +/− 0.00). More largely populated East (blue bars; 6.67 +/− 0.189) and North African (orange bars; 6.36 +/− 0.31) groups almost only consisting of local mentorship pairings also generally indicated agreement with this statement, with East Africa being slightly higher in general agreement. When specifically addressing the IGMP’s impact of understanding of global health security priorities and initiatives, East African respondents indicated relatively higher agreement (6.67 +/− 0.142) in comparison to similar levels observed by North African (6.273+/− 0.359) and West and Southern African respondents (6.33 +/− 0.33). Mentees in West and Southern Africa indicated lower agreement that participation in IGMP helped them connect, interact, and share best practices with other professionals in their region (5.67 +/− 0.33), which is likely due to lack of available mentors in this region. This perspective differs from the vast majority of participants from North (6.45 +/− 0.36) and East (6.67 +/− 0.19) Africa who have completed the program with a mentorship partner within their region, and have comparatively expressed higher agreement with this impact statement. Despite the lack of interaction that the West and Southern African mentees had with local professional networks, they have still expressed interest in engaging with them in the future through their national or regional biosafety association (6.67 +/− 0.33). Similarly, East (6.91 +/− 0.083) and North African (6.54 +/− 0.247) participant responses trended towards strong agreement that they are interested in further engaging with their local biosafety association. This pattern of agreement continued to be observed when considering program impact on the development of leadership and networking skills to interact with and influence others in the developing local professional field. However, there was comparatively less agreement amongst North African respondents that their participation in the program may further prepare them to address decisionmakers at the national level (6.18 +/− 0.46), with the average response value indicating agreement. This deviation was not observed across East African (6.83 +/− 0.112) and West and Southern African (6.67 +/− 0.33) respondents, whose average agreement trended towards strong agreement.

**Fig. 3:**
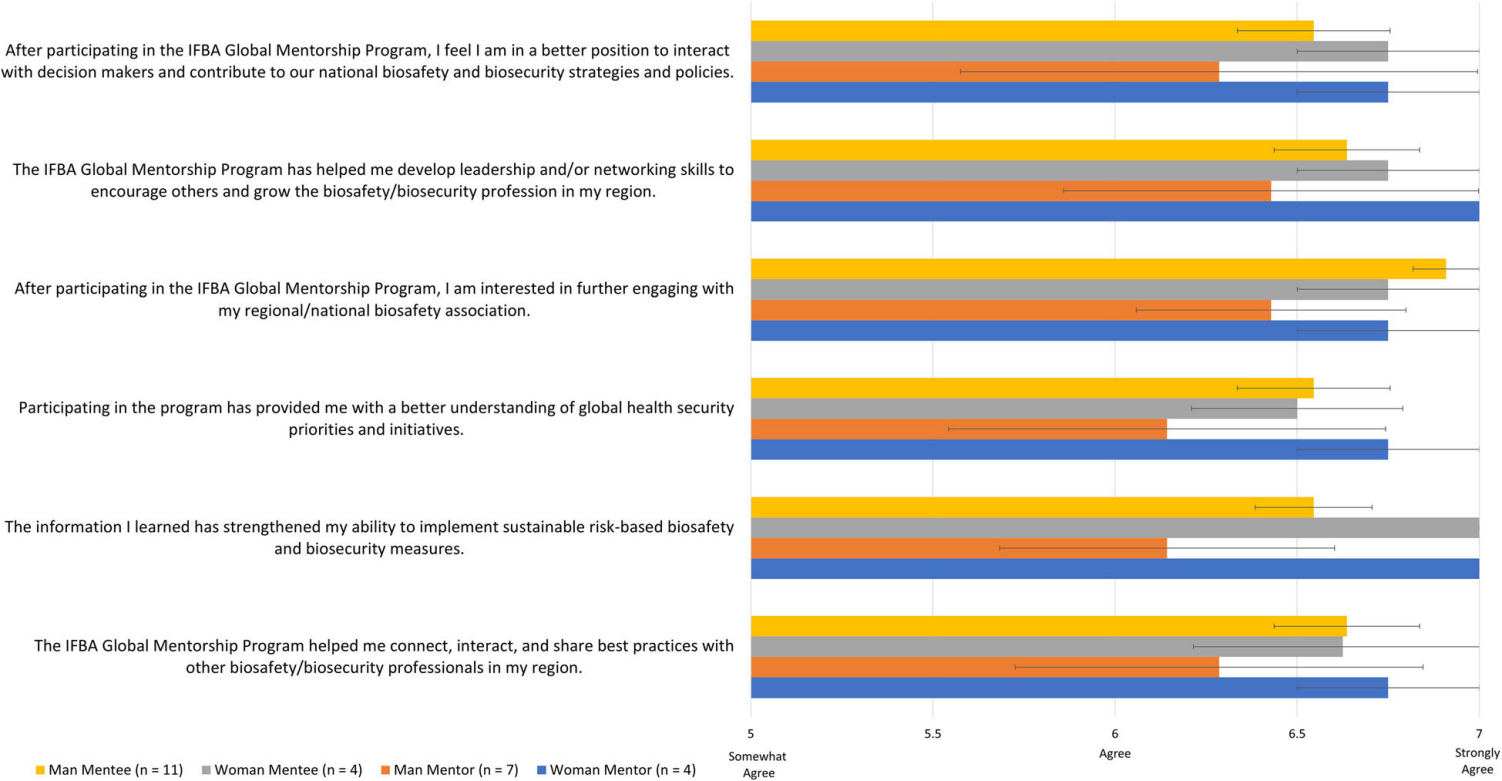
Average exit survey program impact response scores across gender and program role, with standard error. Response scored based upon a 7-point Likert scale, with 1 being strong disagreement with a statement, and 7 being strong agreement

The analysis of program impact statement agreement across participant gender and roles provides an important complement to observed regional perspectives (Fig. 3). Differences across groups of African men and women mentors and mentees showed average overall program impact agreement response to be slightly lower across men participants (6.50 +/− 0.095) compared to women participants (6.73 +/− 0.07), and slightly lower across mentors (6.48 +/− 0.14) compared to mentees (6.63 +/− 0.06). As there were more men mentors (orange bar) than women mentors (blue bar), more women mentees (grey bar) outside of local mentorship pairings, as well as more men respondents overall, gender and role factors at this group’s size remained rather entangled in nature, particularly at the mentee level. The most marked contrast between groups across gender and program role was that between men and women mentors. Average program impact agreement responses amongst women mentors all trended towards strong agreement (6.83 +/− 0.08), with a higher average overall agreement response than women mentees (6.62 +/− 0.18). All women mentors indicated strong agreement with IGMP contributing to the strengthening of their ability to implement sustainable risk-based biosafety and biosecurity measures, as well as to the development of leadership and networking skills to lead and influence their local professional networks.

**Fig. 4:**
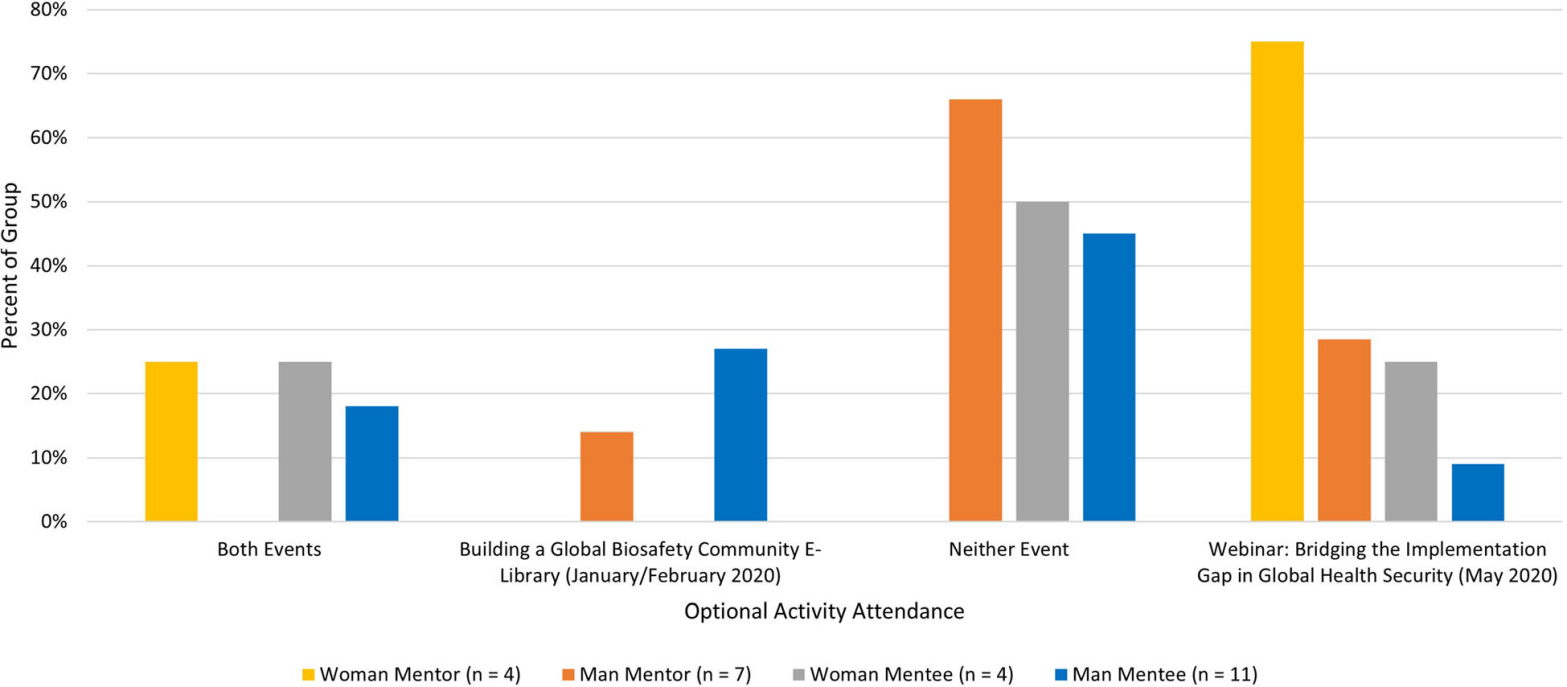
2019–2020 program cycle optional activity attendance proportion across gender and program role (n = 26)

While all survey respondents completed required Progress Reports and related mentorship duties, all women mentor respondents attended at least one optional activity during the 2019–2020 program cycle (Fig. 4, yellow bar), as opposed to 42.86% of men mentor respondents (orange bar) attending one event, and none attending both. It is observed that men participants attended the event with more active participation (round-table discussion), compared to women’s higher relative attendance of an event with more passive participation (webinar). Men and women mentees (blue and grey bars, respectively) were observed to have similar overall optional activity attendance.

**Fig. 5:**
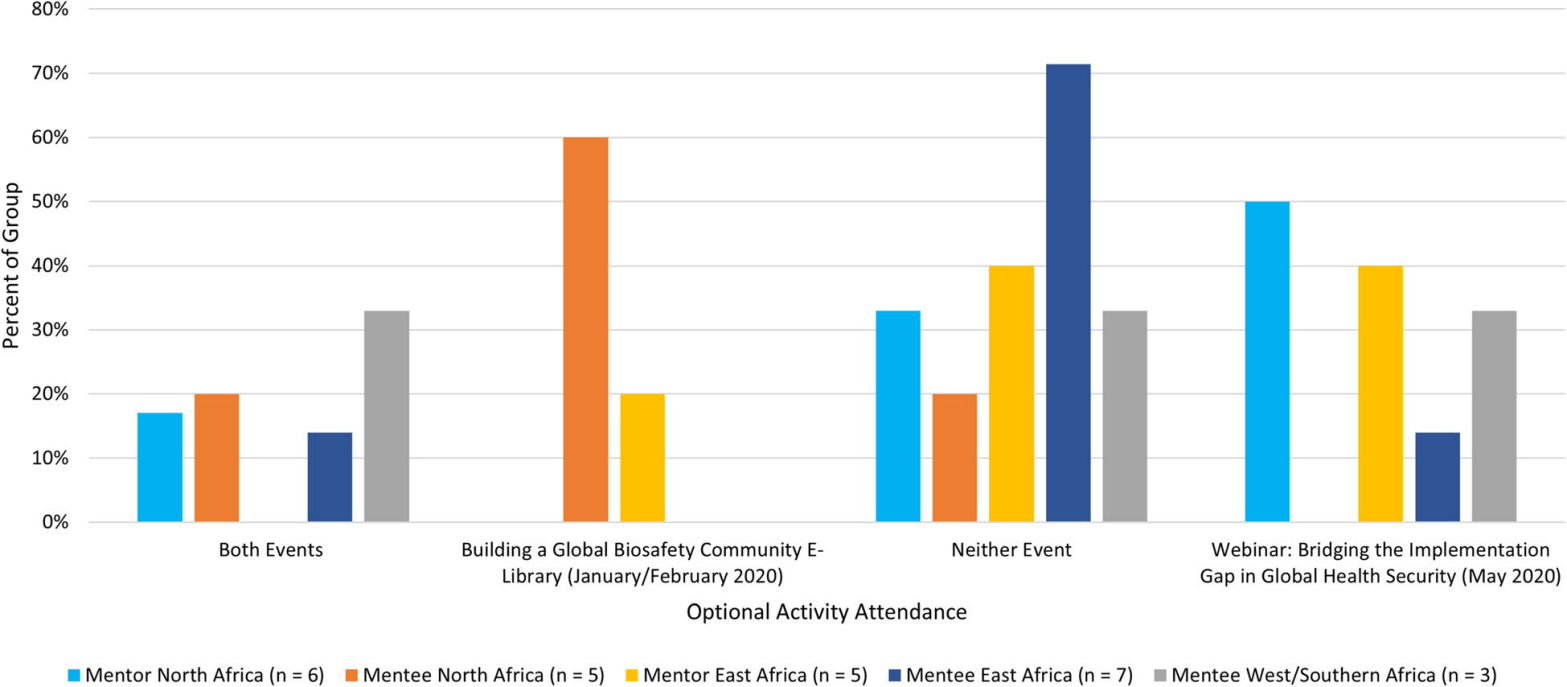
2019–2020 program cycle optional activity attendance proportion across program role and regions (n = 26)

Additional trends were observed across regional comparisons of optional activity attendance. In reference to Fig. 4, it appears that more North African male survey respondents attended the roundtable discussion activity (Fig. 5). East African respondents are observed to have higher relative attendance of the webinar event, but less overall optional activity attendance than North African respondents. The few mentees from West and Southern Africa did not attend either event.

Qualitative responses relevant to program impact were also largely positive, where respondents outlined their perceived benefits of participating in the program with their mentor or mentee. Similar to the quantitative data, there was considerable positive feedback with regards to increased knowledge and understanding of best practices in biosafety and biosecurity, as well as their greater context and applicability to different geographics regions or types of work. Farid*,^1^ a North African mentee in the 2019–2020 program cycle, expressed that, “*[IGMP] was interesting and pertinent, allowing for the knowledge transfer and director learning from my mentor …* ”, who was an experienced biosecurity professional in his region. Many were particularly appreciative of discussion topics directly addressing the COVID-19 pandemic as it quickly gained momentum in early 2020, where considerable work and side projects were conducted by mentorship groups such as national and regional biosecurity gap analysis in relation to the pandemic. Some participants expressed that participation in IGMP at that time was a great source of support as a frontline worker. Michael*, an East African mentee that was also matched with a mentor in his region, explained that they most often discussed, *“…multisectoral and multidisciplinary approaches in handling epidemics and pandemics, [which was just in time for the COVID-19 pandemic], where both my mentor and I are part of national task forces. Discussions were very relevant to the situation*.”

A popular theme amongst feedback submitted by mentors was that of empowerment, where they felt recognized for their expertise in biosafety and biosecurity, and were able to consistently exercise it while working with their mentee(s). John*, an East African mentor, shared that, “*participation in [IGMP] has really made me deeply understand my two certifications in biorisk management and biosecurity. It has … refreshed my mind on the two topics.*” Naima*, a North African mentor, shared that, “*due to [IGMP] and my [IFBA] Professional Certifications, I was [selected] to participate in global training programs and able to collaborate with other scientists regionally and globally*”. Mentors also felt that there was capacity building need for local biosafety and biosecurity programs such as IGMP, and that it would greatly benefit students and young professionals in their respective regions.

Qualitative feedback regarding program impact also directly addressed the development of transdisciplinary human skills used within biosafety and biosecurity professions, as well as policy- and decision-making context to best practices. Sophia*, a South African mentee, shared that, *“[ … my mentor and I] discussed all guiding topics but also discussed processes and procedures carried out in our respective countries regarding biosafety and biosecurity. We discussed legislation, policies, practices, and biosafety cultures. We also discussed resource allocation, funding agencies, networking, and research.*” In addition, certain mentee feedback directly praised their matching with a local mentor. Zenebe*, an East African mentee who was matched with a mentor in his region, stated that his participation in IGMP, “*enable [d] [him] to acquire knowledge, research capacity, confidence, and international exposure*.” He continued to share that, “*[IGMP] is important in making [professional] networks to share experiences and mitigate challenges … [My mentor] clearly understands the circumstances circulating in Africa, [and] particularly in East Africa related to the areas of biosafety and biosecurity … and [addressed] existing [local] infrastructures*.” Other similar feedback expressed that many discussions between mentor and mentee pairs specifically addressed biosafety and biosecurity topics and issues related to their specific country or region, as well as low to middle income countries in general.

## Discussion

### Program impact

The quantitative and qualitative feedback generated by the Exit Survey provides insight into the impacts of a South-to-South biosafety and biosecurity mentorship program across the African continent. A significant outcome of the program was an increased knowledge and understanding of priorities and processes relevant to issues in global health security, including specific acknowledgement of the policy implementation gap. Recurrent themes of the respondents’ reported program benefits included increased professional network building, discussion of best technical practices in their region, and increased motivation to further engage with their national or regional biosafety association following program participation. Translation of concepts to local context included multidisciplinary and multisectoral approaches in recognition of regional and global policy- and decision-making platforms and their work, as well as adaptive translation of best technical practices to pandemic response in low-capacity environments. This integration of technical competency and transdisciplinary human skills through mentorship has been reported to strengthen participants’ abilities to understand technical standard development and their application using risk-based approaches, preparing them to address and collaborate with policy- and decisionmakers in their region.

Exploratory quantitative assessment of Exit Survey responses showed overall positive feedback with regards to program impact statement response scores. The overall reported agreement with these statements amongst African participants indicates their perceived impact of program participation relevant to discussed technical topics and skill development. Regional differences highlight slightly lower scores across North African respondents in comparison to overall scores, as well as the similarly sized East African group’s scores. Interestingly, this trend was inverted with regards to Optional Activity attendance, where there was much higher attendance of North African mentees relative to East African mentee attendance. Regional differences are further elucidated by disaggregating data by gender and program role, where the majority of women mentors were North African and submitted considerably high program impact response scores. Group differences across program role and gender are striking when comparing women mentors and men mentors, where the former group reported higher overall program impact. Feedback from women mentors highlighted mentorship as a mechanism to recognize local biosafety and biosecurity champions, to develop leadership and networking skills relevant to local biosafety and biosecurity professional networks, as well as an opportunity for professional mobilization by providing accessible opportunities for leadership and inclusion in higher level, policy-focused transnational networks. As IGMP recruitment is de-institutionalized and on an individual basis, underrepresented groups within regional and global biosafety and biosecurity networks such as women and youth may have more equitable opportunity for professional development as opposed to technical training session invitations that are often distributed upon consideration of institutional management or executive. The de-institutionalized aspect of IGMP additionally serves to mitigate previously discussed surplus of demand for accessible mentorship for youth in science disciplines across many African regions, ensuring locally relevant support that may have similarly been received within an African mentee’s local institution. Alongside the reported benefits of local mentorship in relation to translation to local context and capacity, South-to-South mentorship equips biosafety and biosecurity champions with an enhanced understanding of their respective health security landscapes. As such, their mentorship may be immersive and integrative, leveraging existing professional connections and lived experience to impart a more complete view of the local health security landscape and its connection to the greater region and world. This specific result exemplifies the importance of discussed human elements of biosafety and biosecurity, where mentees complete the program not only with technical knowledge, but also with the contextual knowledge of local sectors, actors, and events which shape their local health security landscape and implicate them as emerging biosafety and biosecurity specialists. Where mentorship is conducted over a long-term basis and is personal in nature, many mentees indicated that their working relationship with their mentor greatly facilitated their learning, where trust and friendship grew over the course of the program cycle to allow for more detailed discussion of mentee goals and challenges. It must also be noted that the participants who were not matched with a local mentor did have an overall positive experience as well, and still greatly benefit from participating in the program. Where it was observed that participation in IGMP motivated participants to further engage with their National or Regional Biosafety Associations, as well as become more confident conveying their specialized knowledge to their regions’ policy- and decisionmakers, they are propelled towards the harmonized top-down, bottom-up approach to implementation of best practices as appropriate to their regular work and local context. Where accessible and community-led approaches to mentorship such as IGMP are observed to empower biosafety and biosecurity professionals to contribute technical expertise by fostering the human elements of their professional competencies, health security landscapes are argued to be more effectively and sustainably maintained across the spectrum of local capacity.

The undeniable possibility of a ceiling effect regarding collected quantitative feedback invites further future investigation of program efficacy, including more detailed quantitative analysis across groups (e.g. gender, program role, region). Increased representation from West and Southern Africa would allow for the further characterization of respective local professional networks, as well as increase opportunity for local mentorship. More broadly, further analysis including program impact findings from other participating regions outside of Africa such as the Indian subcontinent and Southeast Asia would provide further unique perspectives on program impact which may inform future iterations of the program. As such, observed results from African participants must be considered as the valuable regional perspectives that they are, as opposed to a more global set of responses. It must also be noted that the sample size of Exit Survey respondents provides little opportunity for robust quantitative analysis, and provides only a narrow view across entire regions of professionals that could benefit from programming such as IGMP. Despite follow-up attempts for the Exit Survey, achieving a higher response rate was difficult, likely due to the timing of the Survey’s distribution during the onset of the COVID-19 pandemic in early- to mid-2020. Feedback from IGMP’s 2020–2021 program cycle shows considerable opportunity for further investigation of mentorship’s efficacy regarding technical and human skill development and practice. As the evaluation of mentorship approaches can be difficult due to lack of appropriate indicators [11], further exploratory study on the specific impacts of South-to-South biosafety and biosecurity mentorship would be beneficial. In addition, the discussed observations concerning the impact of mentorship on health security landscapes as relevant to biosafety professionals may be promising for related disciplines in science and cross-sectoral policy. Where discussed skills such as cross-sectoral collaboration are applicable beyond specific health security considerations such as within global and diverse national bio-economies, further precise study of the outcomes of programs such as IGMP would be impactful to implicated workforces. It is also worth considering the further investigation of the impacts of South-to-South mentorship in biosafety and biosecurity on entire professional community subnetworks. Where groups such as professional biosafety associations, institutional biological safety departments, and other groups may provide external observatory perspectives on the impact of their members or employees participating in programs such as IGMP, investigations of this nature may more broadly characterize program impact. Mentorship as a normalized and formalized method of professional development in biosafety and biosecurity may be achieved through maintenance of program interaction with the global professional community’s experts and local professionals, as well as demonstrated support from mentors’ and mentees’ institutional management. Support from institutional management, which may include training sessions specifically dedicated to relevant human skills used in mentoring, protected time allocated for mentoring, and other interventions may further encourage potential mentors to apply. Where emphasis has been placed on the cultivation of positive biosafety culture in the maintenance of risk- and evidence-based biosafety and biosecurity systems [7], supporting local mentorship will demonstrate a tangible commitment from institutional management to the maintenance of biosafety and biosecurity culture and programming over the long term.

### Program development

**Fig. 6:**
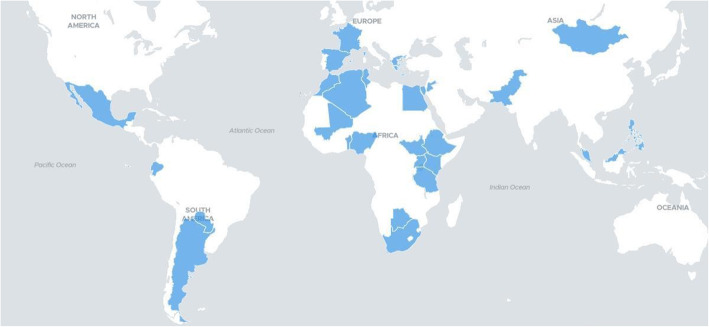
IFBA Global Mentorship Program 2020–2021 program participants geographic distribution (total n = 66). Participants reported residence across 26 countries (Algeria = 5; Benin = 1; Botswana = 1; Ecuador = 1; Egypt = 5; France = 1; Greece = 1; Jordan = 2; Kenya = 7; Malaysia = 2; Mali = 2; Mexico = 2, Mongolia = 1; Morocco = 2; Nigeria = 5; Pakistan = 12; Paraguay = 1; Philippines = 1; South Africa = 1; South Sudan = 2; Spain = 2; Tanzania = 2; Tunisia = 1; Uganda = 4)

Lessons learned from the pilot 2019–2020 program cycle have steered the expansion and delivery of IGMP in its 2020–2021 program cycle. Recruitment for this program cycle involved direct engagement with leaders of regional and national biosafety associations, and encouraging application from their memberships as mentees and mentors alike. Association leaders who applied and were recruited as mentees were matched in an out-of-region South-to-South partnership with experienced mentors that were also regional champions and leaders of their regional associations and professional networks. There was also a similarly involved call for mentor applications to fill previous gaps in West Africa and for providing gender-matched mentors to women mentees. In total, 66 participants from 26 countries were admitted to the 2020–2021 IGMP program cycle. Of those, 37 participants (17 mentors, 20 mentees) from 13 countries are located on the African continent (Fig. 6).

As findings from the 2019–2020 IGMP program cycle highlighted differences in program impact across gender, the subsequent 2020–2021 IGMP program cycle was implemented with the inclusion and empowerment of women in biosafety and biosecurity in mind. Where it was observed that women mentors responded positively to the opportunity to attend interactive Optional Activities, this facet of the program was upscaled to monthly interactive colloquium sessions. During the application period for the 2020–2021 program cycle, there was particular emphasis in the call for applications on the interest of recruiting women mentors and mentees for gender-based and local matching. The desire to recruit more women mentors for the program was advertised to IFBA Member Associations, Observer Organizations, and other stakeholder groups. Additionally, as gender equity in biosafety and biosecurity was identified as a key priority for IFBA, the IFBA Equity-Focused Coordinating Committee (IFBA ECC) was developed in 2020 to steer ongoing IFBA programs and projects which includes IGMP [22, 23]. The work of the IFBA ECC, composed of diverse biosafety and biosecurity professionals from around the world, will review the continued implementation of IGMP and ensure that it embodies best practices in diversity, equity, and inclusion as the program continues to grow and develop.

As mentioned, the significant interest in Optional Activities hosted during the 2019–2020 program cycle has resulted in the development of a monthly virtual colloquium series during the 2020–2021 program cycle. Each colloquium session, which matches the monthly discussion topic, features key decisionmakers and champions in regional and global biosafety and biosecurity, allowing them to directly engage with program participants through interactive presentation sessions. IGMP colloquium speakers are composed of professionals from IFBA governance, executive members of IFBA Member and Observer Associations, government agencies, non-governmental organizations, academia, and private industry. The 2020–2021 program cycle’s colloquium series has featured several women speakers with significant leadership experience across professional sectors relevant to biosafety and biosecurity. In addition to supplementing specific knowledge and understanding of given topics, these sessions often result in discussion of translation to local biosafety and biosecurity systems, as well as questions about how one may learn more about a presented topic or get involved in a global or regional biosafety or biosecurity initiative. In this way, these colloquia serve as important hubs of communication between IGMP participants and the global community, as well as between mentorship pairs across regions.

## Conclusion

The integrative approach to professional development of the described South-to-South biosafety and biosecurity mentorship program complements traditional technical training and certification programming through its direct acknowledgement and facilitation of professional human skill development in a locally relevant manner. IGMP’s community led approach and specific positive reported outcomes supporting African health security networks point towards South-to-South mentorship as a sustainable policy implementation tool.

## Supplementary Information

**Additional File 1.** IFBA Global Mentorship Program Sample Discussion Points Newsletter. Description: Sample monthly discussion points newsletter document provided to program participants to discuss foundational and emerging topics in biosafety and biosecurity, with emphasis on local risk-based approaches and human skills development. The attached monthly discussion points newsletter from October 2020 (2020–2021 program cycle) provides an introduction to risk communication, and promotes the IFBA Biosafety Heroes program.

**Additional File 2.** IGMP 2019–2020 mean exit survey program impact response scores with standard error, across region, gender, and program role. Table listing means and corresponding standard error of program impact response scores between groups, including overall group mean program impact scoring across exit survey program impact questions. Completes data highlighted in Results section of manuscript.

## Data Availability

The data that support the findings of this paper are available from the corresponding authors upon request.

## Abbreviations

IFBA: International Federation of Biosafety Associations
IFBA ECC: IFBA Equity-Focused Coordinating Committee
IGMP: IFBA Global Mentorship Program
WHO: World Health Organization
FAO: Food and Agriculture Organization of the United Nations
OIE: World Organization for Animal Health

## Footnotes

1 Pseudonyms used to maintain confidentiality.
