# Supplementary material for: South-to-south mentoring as a vehicle for implementing sustainable health security in Africa
Source: One Health Outlook. 2021 Oct 6;3:20. doi: 10.1186/s42522-021-00050-x (PMC8492092; doi:10.1186/s42522-021-00050-x)
Supplement: Supplementary file 1 — Additional File 1. IFBA Global Mentorship Program Sample Discussion Points Newsletter. Description: Sample monthly discussion points newsletter document provided to program participants to discuss foundational and emerging topics in biosafety and biosecurity, with emphasis on local risk-based approaches and human skills development. The attached monthly discussion points newsletter from October 2020 (2020–2021 program cycle) provides an introduction to risk communication, and promotes the IFBA Biosafety Heroes program. [file 42522_2021_50_MOESM1_ESM.pdf]

International Federation of Biosafety Associations

www.InternationalBiosafety.org

IFBA Global Mentorship Program

Month 3 – October 2020

This Month's Topics:

- An Introduction to Risk Communication
- IFBA Biosafety Heroes Award – Last Call!

Quick Links & Resources:

- IFBA Website
- Fill Out an Online Progress Report
- Outline: ISO35001: Biorisk Management for Laboratories and Other Related Organizations (2019)
- Crisis Emergency Risk Communication in an Infectious Disease Outbreak (CDC)
- Free Course: Risk Communication Essentials (World Health Organization)
- Communicating Risk in Public Health Emergencies (World Health Organization)

For Questions or Concerns, Please Contact: Stephanie Norlock, IFBA Program Officer, s.norlock@internationalbiosafety.org

A Note From the Program Coordinator…

Dear Program Participants,

I hope that you all have been experiencing a positive month as we end the first quarter of the 2020-2021 IFBA Global Mentorship Program cycle. It has been such a joy already communicating with mentorship pairs and learning more about what interests you in the professional realm of biosafety and biosecurity!

Because we will be entering Month 4 of the program next month, I would like to kindly remind mentorship pairs who have not yet submitted a Progress Report online to do so as soon as they are able. As a quick reminder, Progress Reports are the primary means of keeping track of your participation in the program, and there is a minimum submission rate of 1 Progress Report every three months (making 4 minimum total Progress Reports in one year, or the duration of the program cycle). It is important to note that these Progress Reports should be completed with your mentorship partner (your mentor or mentee). If you still aren't sure how to complete a Progress Report, or are having difficulty contacting your mentorship partner, it is important to let me know as soon as possible so that I may assist you.

I would also like to mention that in the coming months, we will soon be starting our Optional Activities for the year. These Optional Activities are different from the external webinars and tools that I have shared with you so far, as they are round table discussions and online lectures that are exclusive to the IFBA Global Mentorship Program. As such, please monitor your email inboxes regularly so that you may reserve a space in the offered activities that you wish to participate in. If you are aware of any topics or speakers that you would like to see featured in these Optional Activities, please feel free to contact me via email.

Thank you for all of your wonderful work so far, and have a fantastic rest of your month!

Stephanie Norlock, IFBA Program Officer

An Introduction to Risk Communication

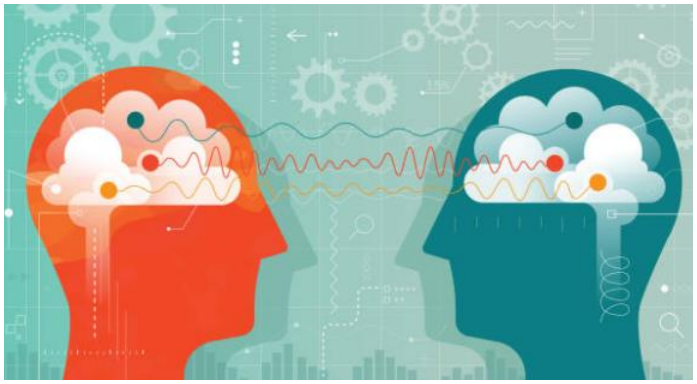

So far in the Mentorship Program, we have partially addressed elements of biological risk assessment, as well as biological risk management. We have also considered gaps and challenges relating to risk management implementation in past mentorship topics, for Mentors that participated in the IFBA Global Mentorship Program last year. When we think about this proper implementation of biosafety and biosecurity guidelines, policies, and frameworks, we may begin with questions and discussion related to capacity and sustainability, such as our topic last month, however, what is the next step after that? How do we as biosafety and biosecurity professionals mobilize the results of our risk assessments and proposed strategies? This introduces the subject of risk communication, which is a crucial, mobilizing element to biological risk management.

Formally, risk communication is considered a component of the traditional tripartite risk analysis paradigm, which also includes risk assessment and risk management. Together, these three elements are responsible for how we perceive, analyze, and ultimately react to various risks through the development and implementation of relevant mitigatory strategies and systems. Risk communication focuses on the exchange and dissemination of information, including how others react to the information that you may give them. This may suggest that risk communication only occurs after we conduct risk assessment and management (eg explaining a new biosafety framework to laboratory workers after it has been drafted and approved by institutional management), but that isn't necessarily the case. Risk communication goes hand in hand with risk assessment and management not always as a logical 'next step', but to help enable or facilitate the assessment and management in the first place. An example of risk communication preceding specific risk assessment or risk management could include technical presentations proposing to increase funding for biological safety officer staffing in a research centre. As such, risk communication should not just be considered within the context of training or as a final step within the process of biorisk management, but as part of a dynamic and multidimensional analysis of risk.

Within the context of general biorisk management, risk communication is specifically addressed in ISO35001, *Biorisk management for laboratories and other related organizations* (2019). The nature of this standard's inclusion of risk communication involves outlining the logistics of risk communication in a laboratory setting, including its impact on worker safety and institutional security. As one may begin to see, there is a delicate balancing act between allowing implicated parties to make informed decisions about safe use of biological materials and maintaining a desired level of informational security. Risk communication is often built into strategies relating to emergency or contingency planning, allowing for clear and concise dissemination of information required for rapid mobilization or, ultimately, containment.

This type of planning involves the establishment of information flow between workers, management, and implicated external stakeholders (when relevant), and is often explicit in the roles and responsibilities associated with proper communication. It is important to consider, then, the value of workers with proven competency in biorisk management filling these positions; professionals with proven relevant competency may more effectively contextualize and further explain presented risks to management or other parties with increased agency to react, or even better, be proactive about future risks before they occur.

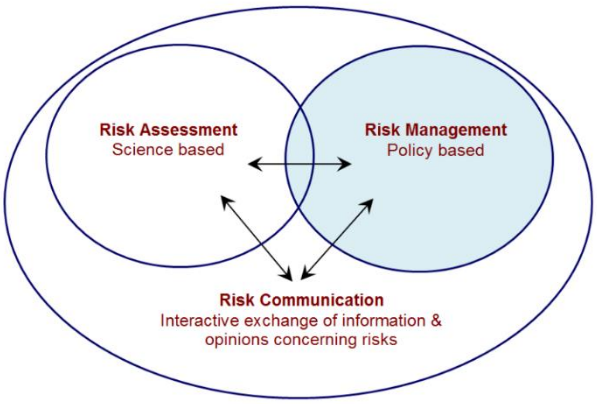

It is also important to note that risk communication relevant to biosafety and biosecurity is not only limited to institutions that work directly with biological materials; risk communication is a vehicle for transdisciplinary influence and change at national governmental and international scales. Professionals such as science advisors and science policy analysts focus on risk communication specifically addressed towards other professionals and colleagues who may not be entirely familiar with the biological or otherwise scientific underpinnings of discussed policy recommendations. A great example of this type of communication is the United Nations Biological Weapons Convention Meeting of Experts, which consists of a series of technical presentations, meetings, and discussions from technical experts in biological and global health security to form recommendations for the United Nations Biological Weapons Convention Meeting of States Parties, which is more largely composed of politicians and other state officials. As mentioned, risk communication within these contexts may allow for expanded or more precise future investigations of risk, where local risk assessment and management may be implemented.

A final note on introductory risk communication is a call for reflection regarding its social nature, and how its utility may unfortunately be partially dependent on who is communicating the perceived risk. Reception and acceptance of risk communication has been studied to a considerable degree, and while there are certain biases that seem more beneficial and logical than not (eg trusting those in positions of expertise or authority more than the average person), some behaviour is less than desirable. Women and other marginalized identities may be overlooked, disproportionately critiqued, or otherwise undermined compared to their colleagues of a majority identity presenting similarly identified risks or potential decisions in similar professional capacities of authority. Risk communication requires bidirectional communication, which is difficult or does not take place when marginalized voices are not considered or included. Women are also part of many groups that have disproportionately low access to information relevant to risk management when considering public health and otherwise medical risks – this is particularly relevant considering the fact that the ongoing COVID-19 pandemic disproportionately affects women. With this in mind, it is critical that risk communication is inclusive, which includes equitable accessibility, adequate representation of marginalized populations in decision-making processes, and continued analysis of potential gaps in information exchange across populations.

After reviewing the attached readings, references and tools, consider the following questions with your mentorship partner:

- While the discussion point article above discussed laboratory risk communication, many of the resources and present attention in the biosafety and biosecurity community are focused on risk communication outside of the laboratory in relation to the ongoing COVID-19 pandemic.
    - What are some unique challenges associated with risk communication in a clinical or public health setting compared to primary research or facilities without public access? Are there unique challenges associated with the laboratory setting that are not present in clinical or public health settings?
    - Misinformation is presently a significant challenge generated prior to or in response to COVID-19 risk communication strategies (and is also an answer to the first question above this one). After reviewing the attached resources on emergency risk communication, how would you approach pandemic misinformation that is popular in your region?
- Reflect upon the connections that can be made between local risk assessment approaches and risk communication. Do you think a local risk assessment approach makes risk communication easier? Does this depend on the audience that you communicate these risks to?

IFBA Biosafety Heroes Award – Last Call!

The International Federation of Biosafety Associations (IFBA) is dedicated to supporting and empowering biosafety and biosecurity professionals from around the world. Each year, the IFBA Biosafety Heroes program identify Biosafety Heroes who have proven themselves to be particularly exceptional in their contributions and work regarding biological risk management. This year marks the tenth anniversary of the IFBA Biosafety Heroes Program, where the first awards were given at the IFBA Meeting in Bangkok, Thailand in February 2011.

IFBA Certified Professional

Click here to review the full nomination criteria for this year's Biosafety Heroes awards, and how to submit a nomination. The deadline for nominations is ***November 1st 2020*** (which is very soon) – as such, if you would like to nominate a potential Biosafety Hero, please do not hesitate!

If you have any questions about the IFBA Biosafety Heroes program, please send an email to the IFBA Secretariat at secretariat@internationalbiosafety.org
