# Supplementary material for: South-to-south mentoring as a vehicle for implementing sustainable health security in Africa
Source: One Health Outlook. 2021 Oct 6;3:20. doi: 10.1186/s42522-021-00050-x (PMC8492092; doi:10.1186/s42522-021-00050-x)
Supplement: Supplementary file 2 — Additional File 2. IGMP 2019–2020 mean exit survey program impact response scores with standard error, across region, gender, and program role. Table listing means and corresponding standard error of program impact response scores between groups, including overall group mean program impact scoring across exit survey program impact questions. Completes data highlighted in Results section of manuscript. [file 42522_2021_50_MOESM2_ESM.docx]

**Table 2.** *IGMP 2019-2020 mean exit survey program impact response scores with standard error, across region, gender, and program role (total n=26). Response scores based upon a 7-point Likert scale, with 1 being strong disagreement with a statement, and 7 being strong agreement. PI1: The information I learned has strengthened my ability to implement sustainable risk-based biosafety and biosecurity measures; PI2: The IFBA Global Mentorship Program helped me connect, interact, and share best practices with other biosafety/biosecurity professionals in my region; PI3: Participating in the IFBA Global Mentorship Program has provided me with a better understanding of global health security priorities and initiatives; PI4: I am interested in further engaging with my regional/national biosafety association; PI5: IGMP has helped me develop leadership and/or networking skills to encourage others and grow the biosafety/biosecurity profession in my region; PI6: I feel I am in a better position to interact with decisionmakers and contribute to my national biosafety and biosecurity policies and/or strategies.*

| Variable | Group Mean | PI1 | PI2 | PI3 | PI4 | PI5 | PI6 |
| --- | --- | --- | --- | --- | --- | --- | --- |
| Region |  |  |  |  |  |  |  |
| East Africa | 6.75 (+/- 0.065) | 6.667 (+/-0.19) | 6.667 (+/-0.14) | 6.667 (+/- 0.22) | 6.917 (+/- 0.083) | 6.750 (+/- 0.18) | 6.833 (+/- 0.11) |
| North Africa | 6.393 (+/- 0.14) | 6.454 (+/- 0.37) | 6.364 (+/- 0.31) | 6.273 (+/- 0.36) | 6.545 (+/- 0.25) | 6.545 (+/- 0.37) | 6.182 (+/- 0.46) |
| West & Southern Africa | 6.500 (+/- 0.14) | 5.667 (+/- 0.33) | 7.00 (0) | 6.333 (+/- 0.33) | 6.667 (+/- 0.33) | 6.667 (+/- 0.33) | 6.667 (+/- 0.33) |
| Gender |  | - |  |  |  |  |  |
| Men | 6.500 (+/- 0.095) |  |  |  |  |  |  |
| Women | 6.730 (+/- 0.070) |  |  |  |  |  |  |
| Role |  |  |  |  |  |  |  |
| Mentors | 6.480 (+/- 0.14) |  |  |  |  |  |  |
| Mentees | 6.630 (+/- 0.060) |  |  |  |  |  |  |
| Gender & Role |  |  |  |  |  |  |  |
| Women Mentors | 6.833 (+/- 0.078) | 6.750 (+/- 0.25) | 7.00 (0) | 6.750 (+/- 0.25) | 6.75 (+/- 0.25) | 7.00 (0) | 6.750 (+/- 0.25) |
| Men Mentors | 6.286 (+/- 0.21) | 6.286 (+/- 0.56) | 6.143 (+/-0.46) | 6.143 (+/-0.60) | 6.429 (+/- 0.37) | 6.429 (+/- 0.57) | 6.286 (+/-0.71) |
| Women Mentees | 6.625(+/- 0.12) | 6.00 (+/-0.41) | 7.00 (0) | 6.500 (+/-0.29) | 6.750 (+/- 0.25) | 6.750 (+/- 0.25) | 6.750 (+/- 0.25) |
| Men Mentees | 6.636 (+/- 0.074) | 6.636 (+/-0.20) | 6.545 (+/- 0.16) | 6.545 (+/- 0.21) | 6.909(+/-0.091) | 6.636 (+/- 0.20) | 6.545 (+/- 0.21) |
